# 3R centres contributions to change animal experimentation: A field report from Charité 3^R^, the 3R centre of a medical faculty

**DOI:** 10.1038/s44319-024-00262-y

**Published:** 2024-09-13

**Authors:** Ida Retter, Laura Behm, Lisa Grohmann, Karin Schmelz, Jennifer Rosowski, Stefan Hippenstiel

**Affiliations:** 1 Charité – Universitätsmedizin Berlin, Corporate Member of Freie Universität Berlin and Humboldt-Universität zu Berlin, Charité 3R, Berlin, Germany; 2 Si-M/‘Der Simulierte Mensch’, Technische Universität Berlin and Charité - Universitätsmedizin Berlin, Berlin, Germany; 3 Charité – Universitätsmedizin Berlin, Corporate Member of Freie Universität Berlin and Humboldt-Universität zu Berlin, Department of Infectious Diseases, Respiratory Medicine and Critical Care, Berlin, Germany; 4 Present Address: VolkswagenStiftung, Hannover, Germany

**Keywords:** Economics, Law & Politics, Methods & Resources

## Abstract

Dedicated 3R centres support and accelerate the transition to non-animal models in biomedical research.

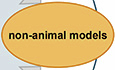

The principle of 3R—to replace, reduce and refine the use of animals in research—was formulated more than 60 years ago (Russell & Burch, 1959). It has been continuously adapted and modified since and found increasing acceptance as the ethical and legal basis for regulating animal experimentation, most prominently in the EU (Parlament & Rat der Europäischen Union). Current laws police the use of vertebrates and cephalopods in particularly, but studies on other animal species show that this demarcation is open to debate (Lenharo, 2024). Notwithstanding, the principle and the laws and regulations based on it, urge scientists to consider alternatives for animal experimentations and to reduce the number of animals whenever possible with the ultimate goal to replace them altogether. However, its application in practice is often not straightforward. To that end, various research institutions have implemented their own policies or created dedicated structures in order to support scientists to implement 3R principles in their work.

“... various research institutions have implemented their own policies or created dedicated structures in order to support scientists to implement 3R principles in their work.”

Charité – Universitätsmedizin Berlin is one of the largest university hospitals in Europe. As a major biomedical research center, Charité uses around 50,000 animals for experiments per year, all vertebrates, about 90% of which are mice. To realize its ethical responsibility towards patients and animals, Charité founded a 3R centre in 2018: Charité 3^R^. Here we describe the goals of Charité 3^R^, the measures that have so far been successfully implemented, and the challenges we face in improving the implementation of the 3Rs within a large medical faculty.

The 3R principle is forward-looking in a comprehensive way: alternative, human-based models to replace animals can generate data that better represent human biology; improved experimental design and reporting of results improves reproducibility and research quality; and refinement reduces stress in animal experiments, thereby improving translatability of the results. This principle is in line with the choice of the best model for a particular research question, be it an animal or a human-based model. On this basis, Charité 3^R^ aims to improve science, to support change in research practices, and to encourage scientists to explore alternative methods.

## Why is the research system so reluctant to change?

Implementation of the 3Rs requires changing models and methods in the lab. Not surprisingly, considering the use of alternative models or even making changes to well-established ones is challenging. First, the establishment of a new experimental model requires considerable methodological expertise, funding and time, which means a lot of costly model development with little expertise and reference data. This tremendous work often is not recognized by high-impact journals and thus less attractive in a system that predominantly measures research success based on publications. From this point of view, refinement is particularly difficult and less attractive to publish. In addition, the description of elaborated refinement in a method section can simply go unnoticed, especially if this is not explicitly discussed elsewhere in the article, which slows down dissemination.

“... the establishment of a new experimental model requires considerable methodological expertise, funding and time, which means a lot of costly model development with little expertise and reference data.”

To make matters worse, the validation of knowledge in previous animal experiments often requires the combination of several complex and costly alternative models. This applies for refinement approaches too, where, for example, the administration of analgesia as well as stress reduction will influence the overall physiology of the animal. The fact that most funders require applicants to have published successfully with the models that are to be used in the proposed project makes it further difficult to establish new models and increases the risk of failure.

For publications, researchers depend on reviewers’ assessment of their work. The reviewers assess whether the models and methods applied can adequately answer the research question often with little weight on ethical considerations. The perceived validity of a specific model in this process heavily depends on the personal experiences and attitudes of the reviewers. However, unlike in regulatory research, there is no formal process to rigorously evaluate whether the applied model is suitable for addressing the research question, and no guarantee that the same model will be deemed sufficient for the next submitted publication.

All this makes 3R very risky, especially for young researchers, who are under time pressure to achieve results and recognition quickly on short-term contracts. These conditions shape the crucial questions: When, why and whether is it appropriate for a research group to establish a new model? Is there a discernible added value for future research, higher-quality publications and the potential for external funding? Will the institution recognize efforts towards the 3Rs, and if so, how? Will a visible commitment to the 3Rs positively influence career paths?

## A holistic 3R implementation approach

**Figure 1:**
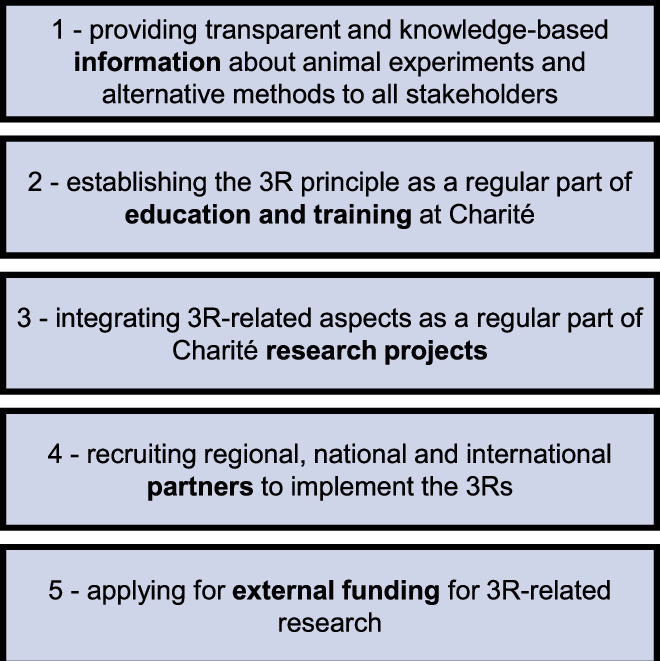
Strategic goals of Charité 3^R^. The five strategic goals of Charité 3^R^ reflect the aim of combining the promotion of 3R with scientific innovation.

**Figure 2:**
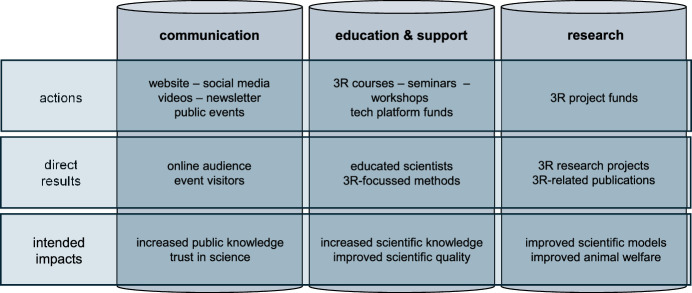
Charité 3^R^ actions and intended impact. Related activities are implemented in 3 different pillars. The direct results of the actions are monitored, while the intended impacts cannot be measured directly.

Charité 3^R^ aims to elevate understanding, diagnosis and treatment of human diseases by supporting a cultural change in biomedical research based on the principles of the 3Rs. It has defined five strategic goals (Fig. 1), which are implemented in the three pillars of Communication, Education and Support and Research (Fig. 2). This tripartite framework mirrors the structure of many 3R centres (Neuhaus et al, 2022a; Neuhaus et al, 2022b), indicating an internationally shared understanding of how to best support the 3Rs.

Communication is strategic goal number one. It is crucial for raising awareness of the 3Rs and promoting the activities and commitment of the Charité faculty. Research stories inform about successful 3R activities at Charité and can inspire further scientists. Furthermore, it is essential to openly inform the public about animal experiments and the current status of implementing alternative methods. This is why we publish the annual animal numbers and provide a direct insight into animal husbandry at Charité via video. Communication with politicians is important to support a political agenda that pushes alternative methods with financial and regulatory mechanisms while ensuring the freedom of science.

“Communication with politicians is important to support a political agenda that pushes alternative methods with financial and regulatory mechanisms while ensuring the freedom of science.”

The pillar ‘Education & Support’ is based on the idea that knowledge dissemination and direct support are key to shift research practices towards new experimental models. We focus on training young scientists in new methods as they will shape future practice. With lectures, hands-on workshops, and exchange platforms they obtain the necessary knowledge, tools and mindset to start a career using alternative methods that are currently underrepresented in most university curricula. Design Thinking workshops, with the team of ReThink3R, develop innovative ideas and tangible solutions in the field of 3Rs and train skills such as critical thinking, collaboration and communication that are essential for future innovative researchers. These courses strengthen participants’ mindset and networks, and we see many of them taking up their career in the context of 3Rs approaches.

Our researchers also asked for better support in implementing refinement, which led to the creation of the refinement task force. This task force not only shares knowledge and advice on the use of refined animal procedures at Charité, but also disseminates these within the Charité research community through courses and an internal data platform with video examples and procedure manuals.

The ‘Research’ pillar supports projects at Charité that implement the 3Rs directly in research practice. This start-up financial support is at the heart of the change process and strategic goal number three: methods and models can only change if researchers work directly on them “with a pipette in their hands”—and this requires funding. Since 2018, Charité has allocated 6 million euros to project funding, and has attracted more than 200 scientists from all relevant research areas. The funding calls focus on different scientific areas and career levels and consider the different needs, levels and impact opportunities. Projects are selected in a competitive evaluation process based on external peer review. For projects that include animal experiments, pre-registration in relevant databases (e.g. (Bert et al, 2019; Heinl et al, 2022; van der Naald et al, 2022)) is mandatory and a study design according to PREPARE and ARRIVE guidelines is expected. All projects are requested to publish open access, which we see as a contributing to disseminating 3R knowledge.

We have also supported larger research networks, which led to the implementation of, for instance, patient-derived stem cells models and the development of new treatment options for rare neurodevelopmental diseases (Inak et al, 2021); human-based kidney models with validated iPSC lines (Batool et al, 2023a; Batool et al, 2023b; Ngo et al, 2022); or a pipeline for tissue collection for research use. Tandem projects brought together young researchers from different backgrounds to develop and publish new models and methods (Hegemann et al, 2023; Huehnchen et al, 2022; Maierhof et al, 2023; Schinke et al, 2021a, b).

## Research infrastructure is key for implementing the 3Rs

3R initiatives typically occur in different locations both spatially and organizationally. Nevertheless, centralized structures can provide significant support, as, for example, central animal facilities. The new building “*Der Simulierte Mensch*” (The simulated human, Si-M, www.si-m.org), part of a collaboration between the Technical University of Berlin and Charité, which is scheduled to open in 2025, will give replacement a new home in Berlin. Utilising the different expertise of the partners, Si-M aims to use new technologies—organs-on-a-chip, bioprinting, multimodal omics technologies or high-throughput analytics—to develop advanced human cell and tissue models while providing access to research infrastructures. Si-M projects aim to deepen the understanding of human health and disease and develop new models and methods as alternatives to animal experimentation. The first two floors of the Si-M building are specifically designed to facilitate meetings and interaction not only between scientists, but also with the wider public by serving as a hub for information, curiosity and critical thinking.

Many of the new models and methods used in replacement are based on human tissue samples as the source for adult stem cells, material for the development of 3D organ models such as organoids or patient-derived tumour spheroids. Despite the close proximity of patient care and research at Charité, there are enormous practical difficulties in obtaining fresh biomaterial for these applications. This hinders both scientific progress and development of alternative methods. Consequently, Charité 3^R^ funds a “Primary Tissue Pipeline”, which provides researchers with human samples from the clinics. The pipeline supports the researchers throughout the entire sample collection process: from obtaining the ethics vote to contacting the donor clinics and setting up logistics for the specific project. In this way, the pipeline makes it easier for research groups to set up human-specific models and links basic researchers and clinicians, which sharpens the research questions.

“Many of the new models and methods used in replacement are based on human tissue samples as the source for adult stem cells, material for the development of 3D organ models such as organoids or patient-derived tumour spheroids.”

Which model can really deliver (translational) progress is an extremely difficult question. In order to promote evidence-based decisions in academic research—beyond classic expert opinions—Charité 3^R^ co-funded the CAMARADES Berlin project, in which researchers find guidance from meta-analyses and systematic reviews of animal experiments as a reduction strategy. The investigation of non-animal-based models and methods, such as organoids, in future meta-analyses and systematic reviews is a crucial step for advancing these developments in a targeted and resource-efficient manner.

The “Experimental Imaging” platform bundles Charité’s small animal in vivo imaging activities and is headed by a Charité 3^R^-funded professorship. Through intensive consulting and training activities, this platform plays a strong multiplier role for 3Rs and enables a reduction in the number of animals through multimodal and longitudinal studies.

Charité 3^R^ is also funding a pilot project to analyse the external validity of a new animal model, the “wildling mice”, which should have a more mature adult immune system with potential higher translational prediction (Rosshart et al, 2019; Villanueva, 2019). In cooperation with the QUEST Center for Responsible Research (https://www.bihealth.org/de/translation/innovationstreiber/quest-center), this project has a strong focus on quality assurance measures.

## Replacement versus refinement

The EU Directive 2010/63 on the use of animals for scientific purposes aims to “achieving the final goal of full replacement (…) as soon as it is scientifically possible to do so” (Parlament & Rat der Europäischen Union). Probably all researchers would agree with this statement. The question is about whether and when it is scientifically possible to do so.

Replacement methods are developing rapidly. Several renowned biomedical research centres are currently developing and using alternative human-based methods, including the Wyss Institute for Biologically Inspired Engineering at Harvard University, USA, or the Institute of Human Biology at Basel, Switzerland. On the basis of this encouraging progress, animal rights activists argue that animal experiments are no longer needed. This opinion is often reinforced by the media and disseminated to the public. Unfortunately, an overestimation of replacement methods seems to hinder sustainable support for refinement measures.

The European citizen initiative “Save cruelty-free cosmetics” for instance makes a complex mixture of claims about regulatory authorities, companies performing animal tests and academic research that may reinforce public impression that all animal experiments are dispensable, and all we need is support to develop replacement methods. If this development guides politicians and research funders to assign less resources for refinement measures, the successful lobbying of animal protection activists absurdly causes less animal protection in practice. Considering the fact that more than 8 million animals are still used in experiments in Europe every year (https://environment.ec.europa.eu/topics/chemicals/animals-science/statistics-and-non-technical-project-summaries_en), a pragmatic approach must consider both, refinement and replacement.

## Funding is key—but 3R centres go further

Researchers are very creative in adapting to funding opportunities. Therefore, we are convinced that funding calls influence the way researchers plan their projects. Research funders can affect the methods and frameworks of research projects by topic-specific calls or specific funding conditions. The current boost of microphysiological systems is based on excellent and hard-working researchers, but also on substantial funding provided by governmental agencies and private donations. Efforts such as the NIH’s Complement Animal Research In Experimentation (Complement-ARIE) Programme are designed to hasten the creation, standardisation, validation, and application of human-centric New Approach Methodologies (NAMs). With an expected investment of US$ 390 million by 2034, these long-lasting initiatives will have an enormous impact (https://commonfund.nih.gov/complementarie).

**Figure 3:**
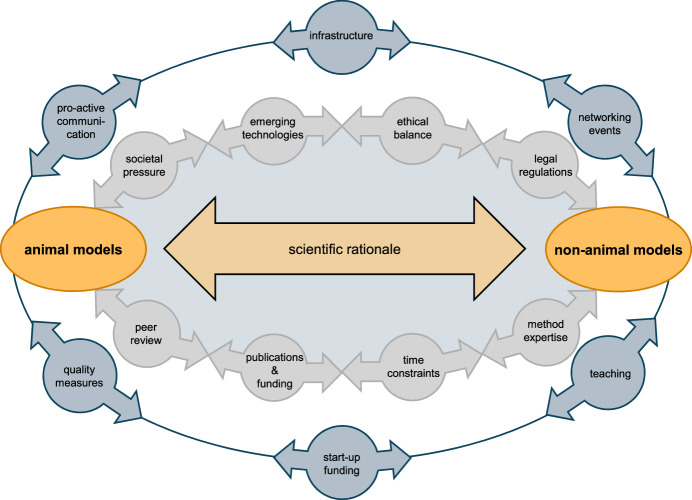
3R centres accompany the change process in academic biomedical research. The decision to use a particular scientific model is, and should be, driven primarily by the scientific needs, provided that an animal experiment includes a harm-benefit assessment. Several pull and push factors inherent to the scientific system (grey), such as advancing technologies, societal pressures or methodological core competencies, influence this decision and shape the change process in biomedical research. 3R centres accompany and mediate this change process by diverse measures (blue).

However, the mission of 3R centres goes beyond research funding. They accompany the ongoing change process in academic biomedical research (Fig. 3). Animal-based research requires different infrastructure and staff than animal-free methods. Consequent promotion of animal-free methods means, with the same financial resources, less support for animal-based research. This foreseeable re-allocation of money can cause substantial resistance in the affected institutions. In particular, as animal welfare officers and caretakers, who are part of the animal-based research infrastructure and often engaged in the local 3R centres, are the main drivers of refinement and reduction measures. They play an essential role in guiding academic research towards fewer and better experiments.

“... animal welfare officers and caretakers, who are part of the animal-based research infrastructure and often engaged in the local 3R centres, are the main drivers of refinement and reduction measures.”

In the current situation, they need not fear for their jobs; on the contrary, qualified and motivated staff are urgently needed. The 3R centres build the common ground for these specialists on animal welfare, the replacement specialists—who often used to or still apply animal experiments in parallel—and the research quality specialists, who can advance both animal experiments and alternative methods. In 3R centres these different experts come together and work collaboratively to implement the 3Rs across the board (Harrison, 2024). We conclude that, as long as animals are required for scientific purposes, the work of 3R centres and the sustainable support for all 3Rs is essential—for the sake of research progress and integrity, animal ethics and societal acceptance of biomedical research.

## Supplementary information

Peer Review File

## References

[CR1] Batool L, Raab C, Beez CM, Hariharan K, Kurtz A, Gollasch M, Rossbach B (2023a) Generation of human induced pluripotent stem cell line (BCRTi006-A) from a patient with focal segmental glomerulosclerosis disease. Stem Cell Res 69:103070 36958215 10.1016/j.scr.2023.103070

[CR2] Batool L, Raab C, Beez CM, Kurtz A, Gollasch M, Rossbach B (2023b) Generation of human induced pluripotent stem cell line (BCRTi007-A) from urinary cells of a patient with autosomal dominant polycystic kidney disease. Stem Cell Res 69:103071 36947994 10.1016/j.scr.2023.103071

[CR3] Bert B, Heinl C, Chmielewska J, Schwarz F, Grune B, Hensel A, Greiner M, Schonfelder G (2019) Refining animal research: the Animal Study Registry. PLoS Biol 17:e3000463 31613875 10.1371/journal.pbio.3000463 PMC6793840

[CR4] Harrison C (2024) 3R centers tap into the human mindset to bolster replacement, reduction and refinement uptake. Lab Anim 53:166–169 10.1038/s41684-024-01396-1 38956356

[CR5] Hegemann N, Bintig W, Perret PL, Rees J, Viperino A, Eickholt B, Kuebler WM, Höpfner M, Nitzsche B, Grune J (2023) In-ovo echocardiography for application in cardiovascular research. Basic Res Cardiol 118:19 37193927 10.1007/s00395-023-00989-0 PMC10188421

[CR6] Heinl C, Scholman-Végh AMD, Mellor D, Schönfelder G, Strech D, Chamuleau S, Bert B (2022) Declaration of common standards for the preregistration of animal research—speeding up the scientific progress. PNAS Nexus 1:pgac016 36712788 10.1093/pnasnexus/pgac016 PMC9802105

[CR7] Huehnchen P, Schinke C, Bangemann N, Dordevic AD, Kern J, Maierhof SK, Hew L, Nolte L, Körtvelyessy P, Göpfert JC et al (2022) Neurofilament proteins as a potential biomarker in chemotherapy-induced polyneuropathy. JCI Insight 7:e154395 35133982 10.1172/jci.insight.154395 PMC8986065

[CR8] Inak G, Rybak-Wolf A, Lisowski P, Pentimalli TM, Jüttner R, Glažar P, Uppal K, Bottani E, Brunetti D, Secker C et al (2021) Defective metabolic programming impairs early neuronal morphogenesis in neural cultures and an organoid model of Leigh syndrome. Nat Commun 12:1929 33771987 10.1038/s41467-021-22117-z PMC7997884

[CR9] Lenharo M (2024) Do insects have an inner life? Animal consciousness needs a rethink. Nature 629:14–15 38653828 10.1038/d41586-024-01144-y

[CR10] Maierhof SK, Schinke C, Cernoch J, Hew L, Bruske LP, Fernandez Vallone V, Fischer K, Stachelscheid H, Huehnchen P, Endres M et al (2023) Generation of an NCS1 gene knockout human induced pluripotent stem cell line using CRISPR/Cas9. Stem Cell Res 73:103253 37984032 10.1016/j.scr.2023.103253

[CR11] Neuhaus W, Reininger-Gutmann B, Rinner B, Plasenzotti R, Wilflingseder D, De Kock J, Vanhaecke T, Rogiers V, Jírová D, Kejlová K et al (2022a) The rise of three Rs centres and platforms in Europe. Alter Lab Anim 50:90–120 10.1177/02611929221099165 35578444

[CR12] Neuhaus W, Reininger-Gutmann B, Rinner B, Plasenzotti R, Wilflingseder D, De Kock J, Vanhaecke T, Rogiers V, Jirova D, Kejlova K et al (2022b) The current status and work of three Rs centres and platforms in Europe. Alter Lab Anim 50:381–413 10.1177/02611929221140909 36458800

[CR13] Ngo TTT, Rossbach B, Sébastien I, Neubauer JC, Kurtz A, Hariharan K (2022) Functional differentiation and scalable production of renal proximal tubular epithelial cells from human pluripotent stem cells in a dynamic culture system. Cell Prolif 55:e13190 35102634 10.1111/cpr.13190 PMC8891564

[CR14] Parlament E, Rat der Europäischen Union Directive 2010/63/EU. Official Journal of the European Union

[CR15] Rosshart SP, Herz J, Vassallo BG, Hunter A, Wall MK, Badger JH, McCulloch JA, Anastasakis DG, Sarshad AA, Leonardi I et al (2019) Laboratory mice born to wild mice have natural microbiota and model human immune responses. Science 365:eaaw4361 31371577 10.1126/science.aaw4361 PMC7377314

[CR16] Russell WMS, Burch RL (1959) The principles of humane experimental technique. Methuen

[CR17] Schinke C, Fernandez Vallone V, Ivanov A, Peng Y, Körtvelyessy P, Nolte L, Huehnchen P, Beule D, Stachelscheid H, Boehmerle W, Endres M (2021a) Dataset for: Modeling chemotherapy induced neurotoxicity with human induced pluripotent stem cell (iPSC)-derived sensory neurons. Data Brief 38:107320 34485650 10.1016/j.dib.2021.107320 PMC8408513

[CR18] Schinke C, Fernandez Vallone V, Ivanov A, Peng Y, Körtvelyessy P, Nolte L, Huehnchen P, Beule D, Stachelscheid H, Boehmerle W, Endres M (2021b) Modeling chemotherapy induced neurotoxicity with human induced pluripotent stem cell (iPSC)-derived sensory neurons. Neurobiol Dis 155:105391 33984509 10.1016/j.nbd.2021.105391

[CR19] van der Naald M, Chamuleau SAJ, Menon JML, de Leeuw W, de Haan J, Duncker DJ, Wever KE (2022) Preregistration of animal research protocols: development and 3-year overview of preclinicaltrials.eu. BMJ Open Sci 6:e100259 10.1136/bmjos-2021-100259 PMC8928250 35372701

[CR20] Villanueva MT (2019) Lab mice go native. Nat Rev Drug Discov 18:745 31570840 10.1038/d41573-019-00145-1

