# Supplementary material for: 3R centres contributions to change animal experimentation: A field report from Charité 3R, the 3R centre of a medical faculty
Source: EMBO Rep. 2024 Sep 13;25(10):4105–9. doi: 10.1038/s44319-024-00262-y (PMC11467298; doi:10.1038/s44319-024-00262-y)
Supplement: Supplementary file 1 — Peer Review File [file 44319_2024_262_MOESM1_ESM.pdf]

EMBO reports

3R centres contributions to change animal experimentation

Ida Retter, Laura Behm, Lisa Grohmann, Jennifer Rosowski, Karin Schmelz, and Stefan Hippenstiel

Corresponding author(s): Stefan Hippenstiel (stefan.hippenstiel@charite.de) , Ida Retter (retter@volkswagenstiftung.de)

Review Timeline:

Submission Date:	28th Mar 24
Editorial Decision:	10th Apr 24
Revision Received:	27th Jul 24
Accepted:	5th Sep 24

Editor: Holger Breithaupt

Transaction Report: This article was editorially reviewed.
